# Scaling Surface-Guided Radiation Therapy to Larger Lung Cancer Cohorts: Frameless Immobilization and Enhanced Setup Accuracy

**DOI:** 10.3390/life16030517

**Published:** 2026-03-20

**Authors:** Jang Bo Shim, Jeongeun Hwang, Sun Myung Kim, Yeong Cheol Lee, Eun Hee Jeon, Hakyoung Kim

**Affiliations:** 1 Departments of Radiation Oncology, Korea University Guro Hospital, Korea University College of Medicine, Seoul 02841, Republic of Korea; jjankola@hanmail.net (J.B.S.);; 2 Departments of Medical Physics, Kyonggi University, Seoul 16227, Republic of Korea; 3 Department of Medical IT Engineering, Soonchunhyang University, Asan-si 31538, Chungcheongnam-do, Republic of Korea; hwangje02@sch.ac.kr

**Keywords:** surface-guided radiation therapy, lung cancer, intrafractional geometric stability, setup accuracy

## Abstract

**Objectives:** This study aimed to evaluate the setup accuracy and intrafractional geometric stability of surface-guided radiation therapy (SGRT) under frameless immobilization in lung cancer radiotherapy and to assess its clinical utility in a relatively large patient cohort. **Materials and Methods:** A total of 678 treatment fractions from 52 patients with primary non-small cell lung cancer (NSCLC), treated between October 2024 and November 2025, were retrospectively analyzed. Patient setup was performed using SGRT with the Identify system, and cone-beam computed tomography (CBCT) served as the reference for internal target localization Intrafractional setup displacements, center-of-mass (COM) shifts, residual setup errors, and intrafractional clinical target volume (CTV) variations were evaluated. Spatial agreement between planned and intrafractional tumor volumes was quantified using the Dice Similarity Coefficient (DSC). **Results:** The mean CBCT-based intrafractional shifts were −0.01 mm (vertical), 0.03 mm (longitudinal), and 0.01 mm (lateral), indicating negligible systematic errors. The greatest variability was observed in the longitudinal direction (standard deviation, 1.32 mm), with a maximum displacement of 4.58 mm. COM-based analysis demonstrated near-zero mean displacements in all directions, with standard deviations ranging from 0.01 to 0.02 mm. DSC values ranged from 0.91 to 0.98, with a mean of 0.96, indicating excellent spatial agreement between planned and intrafractional tumor volumes. Residual setup errors were predominantly within ±1 mm, and the mean intrafractional CTV volume change was minimal (0.27 cm^3^). **Conclusions:** SGRT-based frameless lung cancer radiotherapy demonstrated high setup accuracy and robust intrafractional geometric stability. Although slightly greater variability was observed in the longitudinal direction, overall positional deviations and volumetric changes remained within clinically acceptable limits. These findings support the feasibility of integrating SGRT with CBCT-guided radiotherapy and suggest potential benefits for workflow efficiency and planning target volume margin optimization.

## 1. Introduction

Accurate patient setup and reproducibility are critical determinants of treatment quality in radiation therapy, particularly for thoracic malignancies such as lung cancer. Respiratory-induced motion of the lung parenchyma and chest wall can result in substantial variations in dose distribution, potentially compromising target coverage and increasing normal tissue exposure. Conventional patient setup approaches typically rely on permanent skin tattoos and laser-based alignment systems. Although these methods provide basic positional guidance, they are inherently limited by intrafractional variability, prolonged setup times, and patient discomfort or dissatisfaction associated with permanent markings.

Surface-Guided Radiation Therapy (SGRT) has emerged as a non-invasive, marker-free alternative that addresses many of the limitations of conventional setup techniques [1,2,3,4,5,6,7,8,9]. By capturing high-resolution three-dimensional surface data, SGRT enables six-degree-of-freedom (6DoF) corrections, including both translational and rotational adjustments, and allows continuous real-time monitoring of patient position throughout treatment delivery. This capability improves initial setup accuracy and facilitates intrafractional motion management, thereby enhancing treatment precision and safety. In breast radiotherapy [10,11,12,13,14,15,16,17], SGRT has been successfully integrated into deep inspiration breath-hold (DIBH) workflows, where consistent respiratory control is essential for minimizing cardiac and pulmonary dose. These studies have demonstrated not only clinical efficacy but also improved patient experience through the elimination of permanent tattoos. In addition, advanced SGRT systems are capable of automatically interrupting radiation delivery in response to patient motion, providing an additional layer of safety and quality assurance.

Despite these advantages, evidence supporting the use of SGRT in lung cancer radiotherapy remains relatively limited compared with breast radiotherapy, where the technology has been widely adopted. Early clinical implementation of SGRT in thoracic oncology has primarily focused on stereotactic ablative radiotherapy (SABR) workflows, where high geometric precision is required but the number of treatment fractions is relatively small [18,19,20,21]. The thoracic anatomy and pronounced respiratory motion associated with lung cancer present unique challenges compared to breast radiotherapy, necessitating careful evaluation of SGRT performance in this clinical context. Expanding the cohort size is essential not only to improve the statistical robustness of setup accuracy assessments but also to characterize intrafractional motion patterns across a broader range of anatomical and clinical scenarios.

In this study, we evaluate the clinical utility of SGRT in a cohort of 52 lung cancer patients, focusing on its ability to achieve accurate initial patient setup and robust real-time motion monitoring under frameless immobilization conditions. By extending SGRT-based workflows established in breast cancer radiotherapy to lung cancer treatment, we aim to determine whether comparable setup accuracy can be achieved while improving workflow efficiency and patient safety without reliance on rigid immobilization devices. The findings from this expanded cohort are intended to inform best practices for implementing SGRT in thoracic radiotherapy and to guide optimal planning target volume (PTV) margin design in the presence of substantial respiratory-induced motion.

## 2. Materials and Methods

### 2.1. Patients

A total of 678 treatment records from 52 patients with primary lung cancer who underwent radiotherapy at Korea University Guro Hospital between October 2024 and November 2025 were retrospectively analyzed. All patients were diagnosed with primary non-small cell lung cancer (NSCLC) and received high-dose thoracic radiotherapy targeting the primary lung lesion only. Inclusion criteria were patients with primary NSCLC who underwent radiotherapy for the primary lung lesion using SGRT-assisted setup during the study period and who provided informed consent for the use of non-reimbursed SGRT. This study focused on primary lung lesions as part of an initial clinical evaluation of SGRT implementation. Exclusion criteria included treatment fractions in which cone-beam computed tomography (CBCT) verification images were unavailable, SGRT surface data were incomplete, or treatment interruptions prevented reliable intrafractional evaluation.

This study was conducted following approval by the Institutional Review Board (IRB) of Korea University Guro Hospital (IRB No. 2025GR0447). Due to the retrospective nature of the analysis, the requirement for additional informed consent for study participation was waived. The larger patient cohort allows for more robust assessment of setup reproducibility, intrafractional motion, and clinical feasibility of SGRT in lung cancer radiotherapy, providing stronger statistical validity compared to previous smaller-scale studies.

### 2.2. Radiation Treatment Planning and Immobilization

The prescribed total dose and fractionation schedules were determined based on tumor size, location, and clinical intent. Twenty-two patients with small (≤4 cm), peripherally located lung lesions were treated using SABR, receiving a total dose of 60 Gy delivered in 4–5 fractions (15 Gy × 4 or 12 Gy × 5). Twenty-eight patients were treated with conventionally fractionated radiotherapy, receiving 60 Gy in 20 fractions, while the remaining two patients received a palliative regimen of 30 Gy in 10 fractions.

Patients were immobilized on a shoulder board, and fiducial markers (lead beads) were placed near the tumor site solely for initial localization during CT simulation. Surface imaging was performed in the CT suite using the Identify system (Varian). Following free-breathing practice, CT scans were acquired with a slice thickness of 2 mm for SABR plans and 3 mm for VMAT or IMRT plans.

Respiratory-induced tumor motion was assessed using four-dimensional CT (4DCT) acquired during simulation. The internal target volume (ITV) was delineated based on the 4DCT dataset to encompass tumor motion across all respiratory phases. Although a quantitative distribution analysis of tumor motion amplitudes was not performed in this study, the use of 4DCT ensured that respiratory-induced tumor motion was incorporated into the treatment planning process.

For target delineation in intensity-modulated radiation therapy (IMRT) or volumetric modulated arc therapy (VMAT) planning, the gross tumor volume (GTV) was defined under lung window settings. The clinical target volume (CTV) was generated by expanding the GTV–ITV by 5 mm in all directions, with adjustments made as necessary to respect adjacent normal anatomical structures. The PTV was generated with an additional 5-mm expansion of the CTV. Prescription guidelines aimed to deliver at least 97% of the prescribed dose to 95% of the PTV, with minimum and maximum doses to 1 cc of the PTV maintained between 95% and 107%, respectively. For SABR planning, the GTV–ITV was derived from 4DCT considering respiratory motion, and the PTV was defined with a 5-mm margin around the GTV–ITV. Prescription guidelines required that at least 95% of the PTV receive the prescribed dose. Dose constraints for normal tissues were strictly adhered to: the percentage of lung volume receiving ≥20 Gy was maintained ≤35%, the mean lung dose ≤ 20 Gy, and the maximum doses to the spinal cord and esophagus did not exceed 45 Gy and 60 Gy, respectively.

Treatment planning was conducted using the Eclipse Treatment Planning System version 16.1 (Varian). VMAT with 2–4 arcs was employed for the majority of patients, while select patients were treated with IMRT using five fixed fields and multiple segments. Dose calculations utilized the Anisotropic Analytical Algorithm (AAA) with a calculation grid resolution of 0.25 cm, delivering 6 MV FFF photon beams at a dose rate of 1200 MU/min.

### 2.3. Patient Setup, Surface Imaging, and CBCT Verification

For all patients, the treatment plan—including reference surface contours generated during CT simulation—was imported into the Identify system (Varian Medical Systems) in the treatment room. Prior to clinical use, the surface imaging system underwent vendor-recommended calibration procedures. Daily system checks and periodic quality assurance tests were performed to verify geometric accuracy and camera alignment according to institutional protocols.

Using the system’s real-time optical surface tracking capability, patients were initially positioned on the treatment couch such that the external surface at the treatment site aligned with the planned contours. For SGRT-based surface tracking, the region of interest (ROI) was defined over the anterior thoracic surface surrounding the treatment area. The ROI included relatively rigid anatomical structures such as the sternum and adjacent rib regions while excluding highly deformable regions, including the abdomen, shoulders, and arms. This strategy was intended to minimize respiratory-induced surface deformation and improve tracking stability.

Patients were positioned within a tolerance of ±1 cm in the translational axes (vertical, longitudinal, and lateral) and ±2° in the rotational axes (pitch, roll, and rotation), covering all six degrees of freedom. This tolerance was used as an initial coarse alignment criterion to ensure that the patient surface was sufficiently close to the planned reference position prior to CBCT verification. Final patient positioning was determined using CBCT-based internal anatomy alignment and couch correction.

Following surface-guided setup, CBCT scans were acquired with the imaging volume centered on the PTV to verify internal anatomy and confirm target localization. Any discrepancies between the external surface alignment and internal target position were corrected prior to treatment initiation.

To evaluate geometric agreement between planned and intrafractional tumor volumes, spatial similarity analysis was performed using the Dice Similarity Coefficient (DSC). The DSC was defined as DSC = 2 × (A ∩ B)/(A + B), where A represents the planned tumor volume derived from the planning CT and B represents the corresponding tumor volume segmented on CBCT images. The DSC ranges from 0 (no spatial overlap) to 1 (perfect spatial agreement) and was used as the primary metric for assessing volumetric spatial concordance during treatment.

In addition to the DSC analysis, centroid-based positional descriptors provided by the Identify surface imaging system were recorded. These center-of-mass (COM) values represent the geometric centroid coordinates of the segmented target structures calculated by the system. The COM metrics were analyzed as supplementary indicators of geometric stability between planned and intrafractional tumor volumes.

## 3. Results

### 3.1. Patient and Treatment Characteristics

A total of 52 patients were included in this study. The majority of patients were treated with VMAT, using either 2-arc or 4-arc techniques, while only two patients were treated with IMRT. Most patients received a total prescribed dose of 60 Gy, administered through various fractionation schemes, including SABR (15 Gy × 4 or 12 Gy × 5) and conventionally fractionated radiotherapy (3 Gy × 20), while two patients were treated with a palliative dose of 30 Gy in 10 fractions.

The CTV varied widely among patients, ranging from 7.0 to 311.4 cm^3^, with a mean planned CTV of 115.5 cm^3^. Tumor locations were distributed across all lung lobes, with the right upper lobe being the most common site.

### 3.2. Intrafractional Setup Errors Based on CBCT

The intrafractional setup shifts measured by CBCT are summarized in Table 1. The average shifts were −0.01 mm (Vrt), 0.03 mm (Lng), and 0.01 mm (Lat), indicating minimal systematic bias. However, variability differed among axes, with the longitudinal direction showing the largest spread (STDEV = 1.32 mm), followed by lateral (0.81 mm) and vertical (0.47 mm).

**Figure 1:**
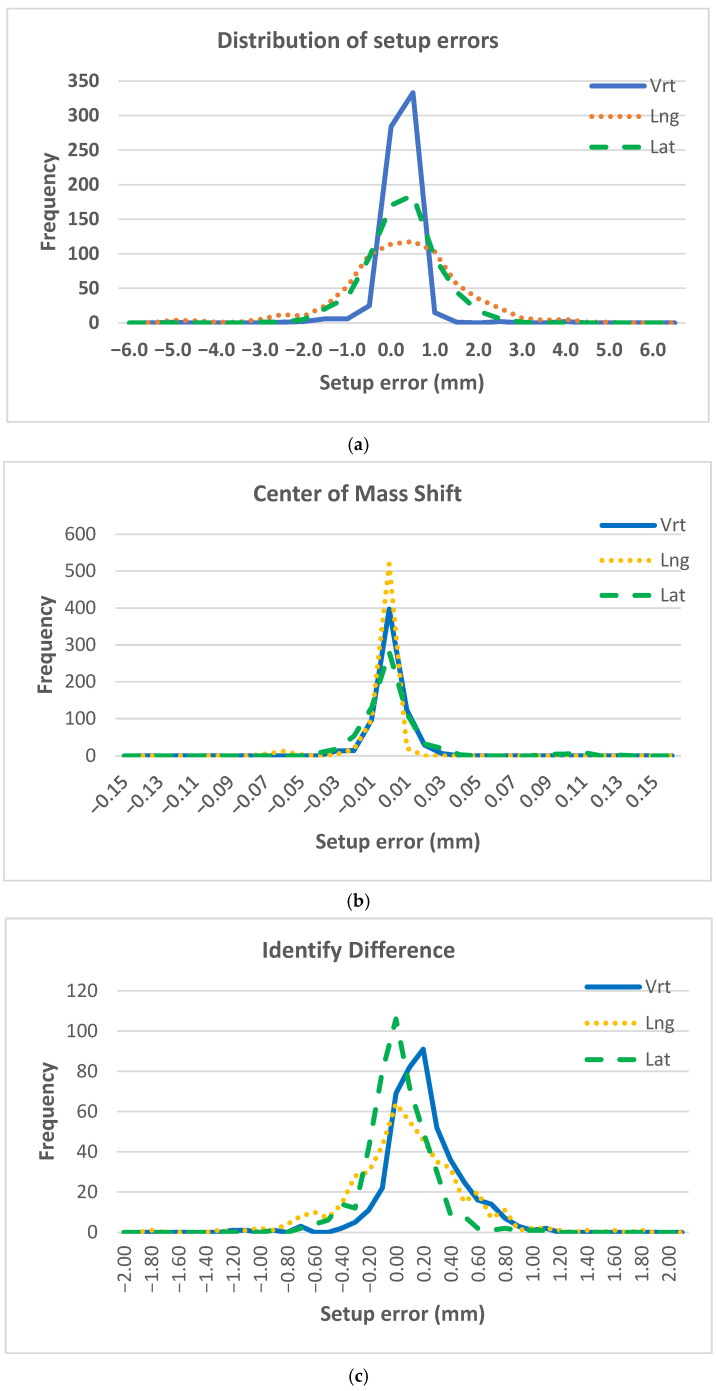
Distribution of intrafractional setup shifts measured using three different metrics: (**a**) CBCT-based setup verification, (**b**) centroid displacement derived from center-of-mass (COM) analysis, and (**c**) residual setup errors reported by the Identify surface imaging system. Distributions are shown for the three translational axes (vertical, longitudinal, and lateral). CBCT, cone-beam computed tomography; COM, center-of-mass; Vrt, vertical; Lng, longitudinal; Lat, lateral.

**Figure 2:**
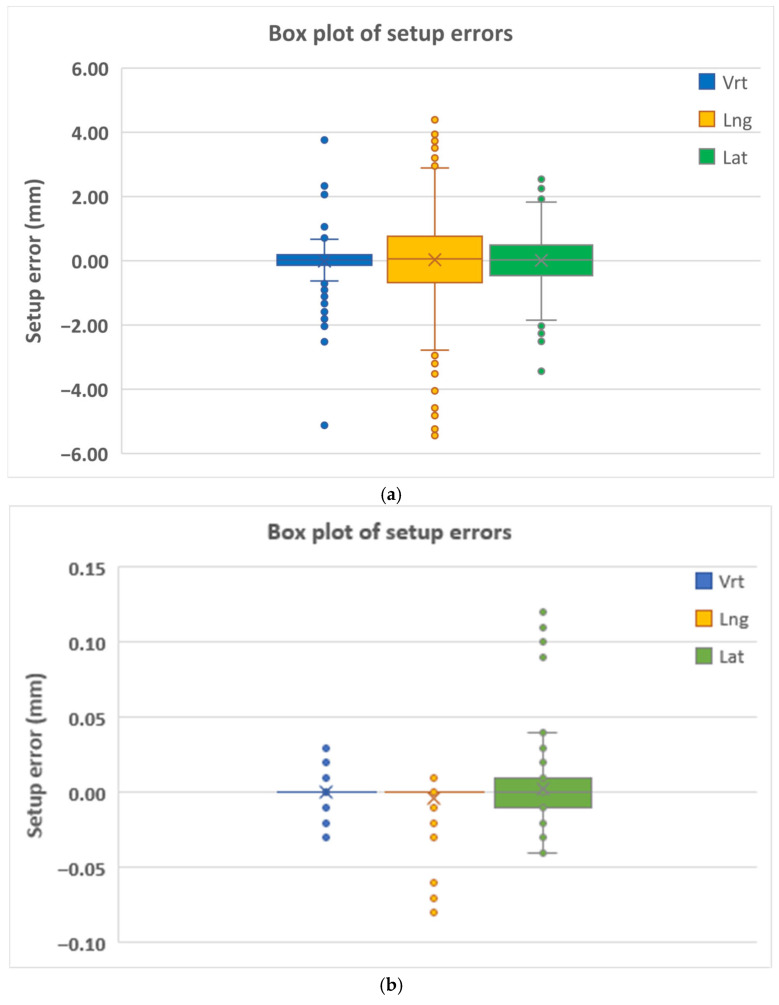

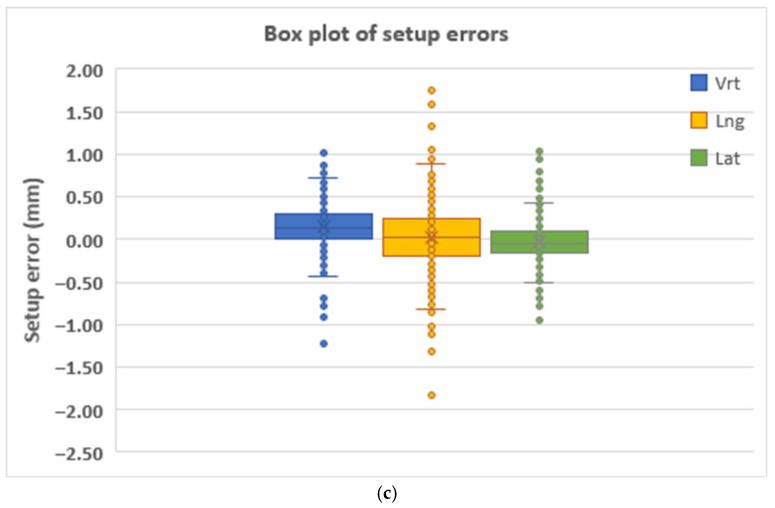
Box-and-whisker plots illustrating the variability of intrafractional setup shifts measured by (**a**) CBCT, (**b**) COM-based centroid displacement, and (**c**) residual setup errors from the Identify system. The plots summarize the median, interquartile range, and outliers for the three translational directions. CBCT, cone-beam computed tomography; COM, center-of-mass; Vrt, vertical; Lng, longitudinal; Lat, lateral.

**Figure 3:**
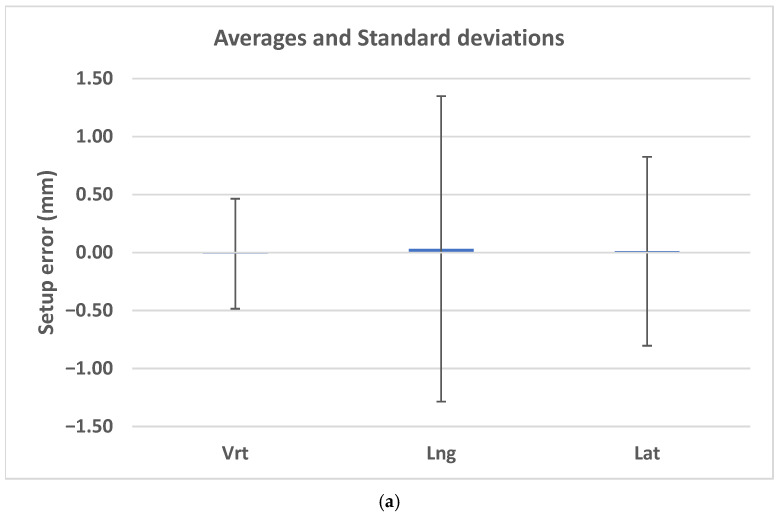

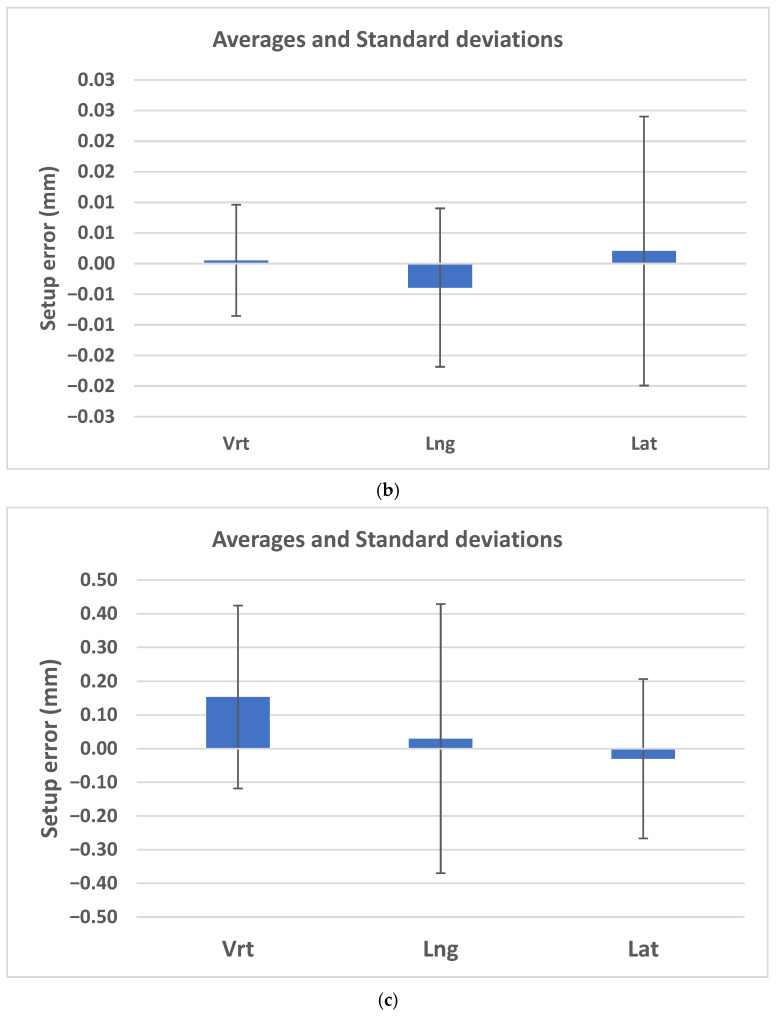
Mean intrafractional setup shifts and corresponding standard deviations for (**a**) CBCT-based measurements, (**b**) COM-based centroid displacement, and (**c**) residual setup errors from the Identify system across the vertical, longitudinal, and lateral directions. CBCT, cone-beam computed tomography; COM, center-of-mass; Vrt, vertical; Lng, longitudinal; Lat, lateral.

The maximum observed shifts reached 4.58 mm in the longitudinal direction and 2.41 mm laterally. Figure 1a demonstrates that most CBCT-based shifts were clustered around zero, while Figure 2a shows wider interquartile ranges in the longitudinal direction. Mean values and standard deviations are illustrated in Figure 3a.

### 3.3. Volumetric Spatial Agreement Between Planned and Intrafractional Tumor Volumes

**Figure 4:**
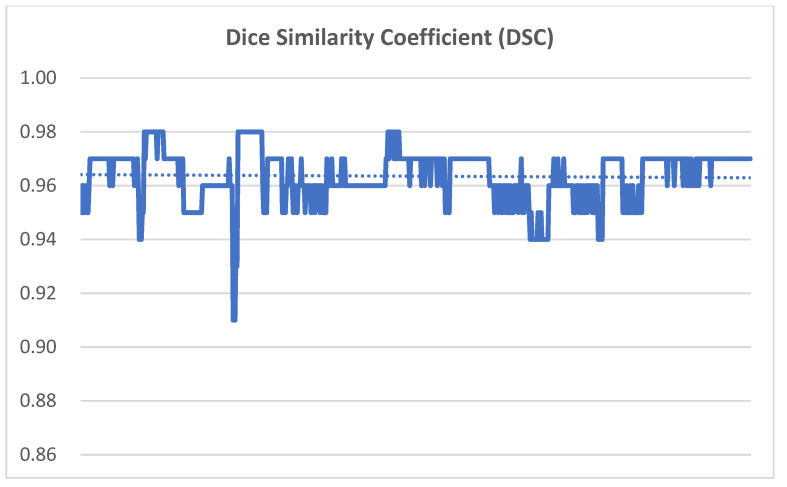
Intrafractional tumor displacement evaluated from CBCT registrations for each patient. Spatial agreement between planned and intrafractional tumor volumes was quantified using the Dice Similarity Coefficient (DSC). CBCT, cone-beam computed tomography.

Spatial agreement between the planned tumor volumes and the corresponding intrafractional CBCT-derived volumes was evaluated using the DSC. The DSC values ranged from 0.91 to 0.98, with a mean value of 0.96, indicating excellent volumetric concordance throughout treatment delivery (Figure 4). These findings suggest that the planned and intrafractional tumor volumes maintained a high degree of spatial overlap during treatment.

The consistently high DSC values across patients indicate strong geometric stability of the target volumes despite small positional variations observed in CBCT-based setup measurements.

### 3.4. Center-of-Mass–Based Positional Descriptors

In addition to the DSC-based volumetric analysis, centroid-based positional descriptors provided by the Identify surface imaging system were evaluated. These COM values represent the geometric centroid coordinates of the segmented target volumes calculated by the system.

The COM analysis demonstrated very small positional variations across fractions, with average shifts of approximately 0.00 mm in all three translational directions and standard deviations of 0.01–0.02 mm. Because the centroid is derived from the spatial distribution of the entire volumetric structure rather than from individual voxel positions, centroid-based calculations may yield sub-voxel numerical precision even when derived from imaging datasets with millimeter-scale voxel resolution. However, these extremely small variations are more likely to reflect the computational characteristics of centroid calculation rather than true anatomical motion at a sub-millimeter scale.

The narrow box plots in Figure 1b, the limited interquartile ranges in Figure 2b, and the minimal standard deviations illustrated in Figure 3b reflect the high numerical stability of centroid-based positional descriptors during treatment. Accordingly, these metrics should be interpreted as indicators of relative geometric consistency rather than absolute measures of physical positioning accuracy.

### 3.5. Identified Setup Errors

The identified setup errors, representing residual discrepancies after correction, showed slightly larger variations than COM descriptors but remained within clinically acceptable limits. The mean residual errors were 0.15 mm (Vrt), 0.03 mm (Lng), and −0.03 mm (Lat), with standard deviations below 0.40 mm in all directions.

The interquartile ranges were largest in the longitudinal direction (0.40 mm), consistent with the CBCT-based observations. Figure 1c and Figure 2c demonstrate that although outliers were present, the majority of residual errors remained within ±1 mm. Mean values and standard deviations are illustrated in Figure 3c.

### 3.6. Intrafractional CTV Changes

**Figure 5:**
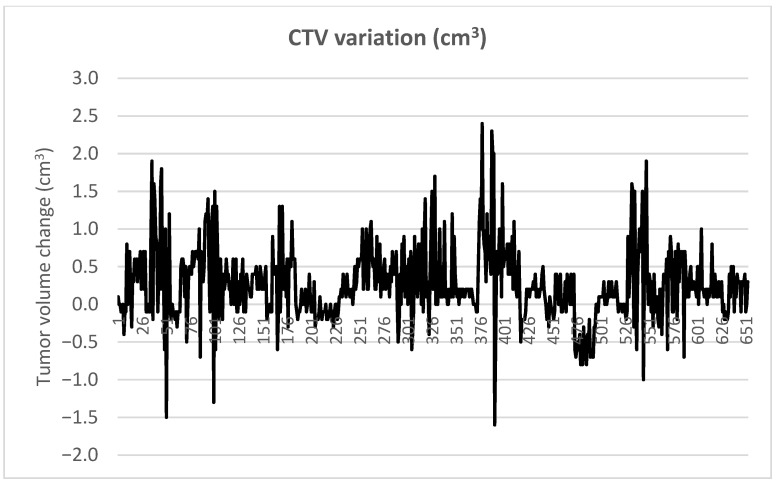
Intrafractional variations in clinical target volume (CTV) relative to the planned CTV during treatment fractions. CTV, clinical target volume.

Intrafractional CTV variations are summarized in Table 2 and Figure 5. The average volume change was 0.27 cm^3^ with a standard deviation of 0.48 cm^3^. This value represents the mean intrafractional volumetric variation calculated per treatment fraction relative to the planned CTV rather than the difference between the cohort mean planned and CBCT-derived CTV volumes.

Although individual cases demonstrated volume changes ranging from −1.5 to +1.9 cm^3^, the median change was only 0.2 cm^3^. The consistently high DSC values across patients further support strong spatial agreement between planned and intrafractional tumor volumes, indicating stable geometric target positioning during treatment.

## 4. Discussion

Several previous studies have investigated the use of SGRT in thoracic radiotherapy, particularly in SABR workflows. Surface-guided positioning has increasingly attracted interest as a non-invasive alternative to conventional tattoo-based alignment methods in modern image-guided radiotherapy. These studies have primarily focused on evaluating setup accuracy by comparing SGRT-derived surface alignment with CBCT-based internal target positioning. Reported translational setup discrepancies are generally within a few millimeters, supporting the feasibility of SGRT-assisted positioning in thoracic treatments.

However, most previous investigations have focused predominantly on surface displacement metrics or setup correction accuracy rather than volumetric agreement between planned and intrafractional tumor geometries. In addition, many studies have been conducted in highly hypofractionated SABR settings involving a limited number of treatment fractions. In contrast, the present study evaluates geometric stability across a broader range of lung radiotherapy workflows, including both SABR and conventionally fractionated treatments, while also incorporating volumetric spatial similarity analysis using the DSC. This complementary evaluation provides additional insight into the stability of target geometry during treatment delivery.

This study systematically evaluated setup uncertainties and intrafractional geometric stability in lung cancer patients treated predominantly with VMAT using frameless immobilization. The analysis incorporated complementary metrics derived from CBCT-based setup verification, volumetric similarity assessment using the DSC, centroid-based positional descriptors (center of mass, COM), and residual setup errors after correction. By analyzing a relatively large cohort, this work provides a comprehensive assessment of geometric stability during thoracic radiotherapy.

CBCT-based setup analysis demonstrated minimal systematic bias, with mean shifts close to zero in all translational directions, confirming the effectiveness of image-guided correction strategies. However, variability differed among axes, with the longitudinal direction exhibiting the largest standard deviation (1.32 mm) and the greatest maximum observed shifts. This pattern is consistent with well-established respiratory-induced motion characteristics in lung tumors, which predominantly occur along the superior–inferior axis. Despite this, the magnitude of these shifts remained within commonly applied PTV margins, suggesting that such variations are unlikely to compromise target coverage in routine clinical practice. The majority of intrafractional CBCT shifts were clustered near zero, indicating stable and reproducible patient setup.

These findings are consistent with previously reported values for SGRT-assisted positioning in thoracic radiotherapy, where translational discrepancies are typically on the order of 1–3 mm. In the present study, setup variability was within a similar or slightly lower range, further supporting the reliability of SGRT-based positioning. Compared with earlier reports, this study extends existing evidence by including a larger cohort and a broader range of fractionation schemes, while also incorporating volumetric analysis using DSC (mean DSC = 0.96). The combined evaluation of positional and volumetric metrics provides a more comprehensive characterization of geometric stability.

In addition to volumetric analysis, centroid-based positional descriptors were evaluated. The COM-based analysis showed extremely small positional variations, with mean shifts near zero and standard deviations of approximately 0.01–0.02 mm. Because centroid positions are derived from the spatial distribution of the entire segmented volume, these values reflect the stability of the geometric center rather than voxel-level positional accuracy. Although centroid calculations can yield sub-voxel numerical precision, such values do not necessarily correspond to true anatomical agreement and should be interpreted with caution. The observed minimal variation is therefore more indicative of numerical stability of the centroid position than clinically meaningful changes in tumor location. Accordingly, COM-based metrics should be considered indicators of relative geometric stability rather than absolute measures of physical positioning accuracy. It should also be noted that SGRT relies on external surface monitoring, and the correlation between surface motion and internal tumor position may vary depending on tumor location, respiratory patterns, and patient-specific anatomy.

Residual setup errors after correction were also minimal, with mean values below 0.15 mm and standard deviations under 0.40 mm in all directions. The majority of residual discrepancies were confined within ±1 mm, supporting the adequacy of currently applied PTV margin strategies even in hypofractionated and SABR-like treatment regimens. The slightly greater variability in the longitudinal direction again highlights the influence of respiratory motion.

Intrafractional CTV volume changes were negligible, with an average variation of only 0.27 cm^3^. Although minor fluctuations were observed in individual fractions, consistently high DSC values indicate that these variations had minimal impact on spatial overlap. This suggests that volumetric deformation during treatment delivery is limited and unlikely to compromise target coverage.

Collectively, these results demonstrate that frameless VMAT-based lung radiotherapy supported by CBCT verification and SGRT can achieve robust geometric stability throughout treatment delivery. The greater variability observed along the longitudinal axis underscores the importance of motion management strategies for respiration-affected tumors. Integration of real-time monitoring approaches, such as SGRT-based motion tracking or time-resolved imaging, may further enhance intrafractional control.

Building upon these findings, the observed level of setup accuracy and intrafractional stability may have implications for PTV margin strategies. Margin design should account for both systematic and random uncertainties, particularly respiratory motion in the superior–inferior direction. In this context, the small residual setup errors observed in this study are consistent with margins commonly used in clinical practice.

However, these findings should be interpreted cautiously. Although robust geometric stability was observed, the results do not directly support margin reduction, as this would require a comprehensive evaluation of additional uncertainties, including interfractional variation, organ deformation, and patient-specific motion characteristics. Nevertheless, the high reproducibility observed across treatment techniques suggests that SGRT may support future investigations into margin optimization. Prospective studies incorporating motion modeling and margin calculation frameworks are warranted.

Several limitations should be acknowledged. This was a retrospective single-institution study, which may limit generalizability. Although larger than many previous reports, the cohort size remains insufficient to capture rare or extreme motion patterns. In addition, COM-based analysis assumes rigid motion and may underestimate localized deformation. While additional analyses were incorporated, the study remains primarily exploratory, and more advanced statistical modeling could provide further insight.

Despite these limitations, the high reproducibility of setup and intrafractional stability suggests that SGRT-assisted positioning can support efficient and reliable image-guided workflows. Improved setup consistency may enable optimized CBCT verification strategies while maintaining geometric accuracy. Furthermore, surface-guided positioning eliminates the need for permanent skin markings and may reduce reliance on additional ionizing imaging during patient alignment, thereby improving patient comfort and potentially reducing staff exposure.

## 5. Conclusions

Thoracic radiotherapy delivered with VMAT under frameless immobilization demonstrated high geometric accuracy when combined with CBCT-guided setup verification and SGRT. Intrafractional CBCT shifts were minimal, and volumetric similarity analysis using the DSC showed strong spatial agreement between planned and intrafractional tumor volumes. Centroid-based positional descriptors (COM) also indicated stable tumor center positions during treatment delivery.

Residual setup errors and intrafractional CTV variations remained within clinically acceptable limits, supporting the adequacy of current PTV margin strategies, including hypofractionated and SABR-like regimens. Although slightly greater variability was observed in the longitudinal direction, overall tumor geometric stability was well maintained, highlighting the importance of appropriate respiratory motion management.

## Figures and Tables

**Table 1:** Statistical data of (a) CBCT setup differences, (b) Center of mass shifts, (c) Identify setup errors.

**(a)** **CBCT setup differences**					
	Vrt		Lng		Lat
**IQR**	**0.29**		**1.34**		**0.85**
Median	−0.12		0.52		0.74
Min	−1.81		−5.26		−1.70
Max	0.81		4.58		2.41
Mode	0.04		−0.01		0.26
**Average**	**−0.01**		**0.03**		**0.01**
**SD**	**0.47**		**1.32**		**0.81**
**(b)** **Center of mass shifts**					
	Vrt	Lng		Lat	DSC
**IQR**	**0.02**	**0.01**		**0.00**	**0.01**
Median	−0.02	−0.01		−0.01	0.96
Min	−0.03	−0.01		−0.01	0.91
Max	0.03	0.01		0.01	0.98
Mode	0.00	0.00		0.00	0.97
**Average**	**0.00**	**0.00**		**0.00**	**0.96**
**SD**	**0.01**	**0.01**		**0.02**	**0.01**
**(c)** **Identify setup errors**					
	Vrt		Lng		Lat
**IQR**	**0.29**		**0.40**		**0.20**
Median	0.34		0.11		0.14
Min	−0.91		−1.83		−0.69
Max	1.08		1.02		0.68
Mode	0.06		0.02		−0.04
**Average**	**0.15**		**0.03**		**−0.03**
**SD**	**0.27**		**0.40**		**0.24**

CBCT, cone-beam computed tomography; Vrt, vertical; Lng, longitudinal; Lat, lateral; DSC, Dice Similarity Coefficient; IQR, interquartile range; Min, minimum; Max, maximum; SD, standard deviation.

**Table 2:** Intrafractional CTV volumetric variation relative to the planned CTV (per treatment fraction).

	Plan Volume (cm^3^)	CBCT Volume (cm^3^)	Difference (cm^3^)
**IQR**	**145.500**	**146.200**	**0.700**
Median	64.10	64.35	0.20
Min	7.00	6.80	−1.50
Max	311.40	312.70	1.90
Mode	102.70	41.60	0.20
**Average**	**115.53**	**96.66**	**0.27**
**SD**	**87.56**	**71.30**	**0.48**

CTV, clinical target volume; CBCT, cone-beam computed tomography; IQR, interquartile range; Min, minimum; Max, maximum; SD, standard deviation.

## Data Availability

The original contributions presented in this study are included in the article. Further inquiries can be directed to the corresponding author.

## Abbreviations

SGRT, surface-guided radiation therapy; 6DoF, six degrees of freedom; DIBH, deep inspiration breath-hold; PTV, planning target volume; NSCLC, non-small cell lung cancer; IRB, Institutional Review Board; SABR, stereotactic ablative radiotherapy; IMRT, intensity-modulated radiation therapy; VMAT, volumetric modulated arc therapy; GTV, gross tumor volume; ITV, internal target volume; CTV, clinical target volume; AAA, Anisotropic Analytical Algorithm; FFF, flattening filter free; MU, monitor unit; CBCT, cone-beam computed tomography; Vrt, vertical; Lng, longitudinal; Lat, lateral; Rtn, rotation; DSC, Dice Similarity Coefficient; COM, center of mass.

## References

[1] Beer K.T. (2022). Introduction of SGRT in clinical practice. Tech. Innov. Patient Support. Radiat. Oncol..

[2] Bry V., Licon A.L., McCulloch J., Kirby N., Myers P., Saenz D., Stathakis S., Papanikolaou N., Rasmussen K. (2021). Quantifying false positional corrections due to facial motion using SGRT with open-face Masks. J. Appl. Clin. Med. Phys..

[3] Liu X., Alexander D., Kayser D., Speck T., Wiersma R.D. (2025). A novel SGRT system for real-time optical surface tracking or guidance. J. Appl. Clin. Med. Phys..

[4] Oliver K., Subick N., Moser T. (2024). A prospective, comparative evaluation of an augmented reality tool (Postural Video) vs. standard SGRT for efficient patient setup. Rep. Pract. Oncol. Radiother..

[5] Paolani G., Strolin S., Santoro M., Della Gala G., Tolento G., Guido A., Siepe G., Morganti A.G., Strigari L. (2021). A novel tool for assessing the correlation of internal/external markers during SGRT guided stereotactic ablative radiotherapy treatments. Phys. Med..

[6] Rudat V., Shi Y., Zhao R., Xu S., Yu W. (2023). Setup accuracy and margins for surface-guided radiotherapy (SGRT) of head, thorax, abdomen, and pelvic target volumes. Sci. Rep..

[7] Song Y., Zhai X., Liang Y., Zeng C., Mueller B., Li G. (2022). Evidence-based region of interest (ROI) definition for surface-guided radiotherapy (SGRT) of abdominal cancers using deep-inspiration breath-hold (DIBH). J. Appl. Clin. Med. Phys..

[8] Sotiropoulou V., Tsironi F., Tolia M., Mazonakis M. (2025). Comparison between the SGRT and the conventional setup method for patients undergoing VMAT for pelvic malignancies. Appl. Radiat. Isot..

[9] Zhao H., Sarkar V., St James S., Paxton A., Su F.F., Price R.G., Dial C., Poppe M., Gaffney D., Salter B. (2024). Verification of surface-guided radiation therapy (SGRT) alignment for proton breast and chest wall patients by comparison to CT-on-rails and kV-2D alignment. J. Appl. Clin. Med. Phys..

[10] Kugele M., Mannerberg A., Norring Bekke S., Alkner S., Berg L., Mahmood F., Thornberg C., Edvardsson A., Back S.A.J., Behrens C.F. (2019). Surface guided radiotherapy (SGRT) improves breast cancer patient setup accuracy. J. Appl. Clin. Med. Phys..

[11] Lai J., Luo Z., Hu H., Jiang L., Wu J., Lei L., Qu L., Wu Z. (2023). SGRT-based DIBH radiotherapy practice for right-sided breast cancer combined with RNI: A retrospective study on dosimetry and setup accuracy. J. Appl. Clin. Med. Phys..

[12] Lastrucci A., Serventi E., Francolini G., Marciello L., Fedeli L., Meucci F., Marzano S., Esposito M., Ricci R. (2024). A retrospective comparison of setup accuracy from CBCT and SGRT data in breast cancer patients. J. Med. Imaging Radiat. Sci..

[13] MacFarlane M.J., Jiang K., Mundis M., Nichols E., Gopal A., Chen S., Biswal N.C. (2021). Comparison of the dosimetric accuracy of proton breast treatment plans delivered with SGRT and CBCT setups. J. Appl. Clin. Med. Phys..

[14] Mankinen M., Viren T., Seppala J., Koivumaki T. (2024). Interfractional variation in whole-breast VMAT irradiation: A dosimetric study with complementary SGRT and CBCT patient setup. Radiat. Oncol..

[15] Rudat V., Shi Y., Zhao R., Yu W. (2024). Setup margins based on the inter- and intrafractional setup error of left-sided breast cancer radiotherapy using deep inspiration breath-hold technique (DIBH) and surface guided radiotherapy (SGRT). J. Appl. Clin. Med. Phys..

[16] Sauer T.O., Ott O.J., Lahmer G., Fietkau R., Bert C. (2023). Prerequisites for the clinical implementation of a markerless SGRT-only workflow for the treatment of breast cancer patients. Strahlenther. Onkol..

[17] Sauer T.O., Stillkrieg W., Ott O.J., Fietkau R., Bert C. (2023). Plan robustness analysis for threshold determination of SGRT-based intrafraction motion control in 3DCRT breast cancer radiation therapy. Radiat. Oncol..

[18] Shim J.B., Kim H., Kim S.M., Yang D.S. (2025). Translating SGRT from Breast to Lung Cancer: A Study on Frameless Immobilization and Real-Time Monitoring Efficacy, Focusing on Setup Accuracy. Life.

[19] Blake N., Pereira L., Eaton D.J., Dobson D. (2021). Surface-guided radiotherapy for lung cancer can reduce the number of close patient contacts without compromising initial setup accuracy. Tech. Innov. Patient Support. Radiat. Oncol..

[20] Guo H.L., Wu W.W., Huan Y., Zhang H.W. (2024). SGRT-based stereotactic body radiotherapy for lung cancer setup accuracy and margin of the PTV. J. Appl. Clin. Med. Phys..

[21] Li Q., Liu R., Han L., Xu Y., Fu H., Nan C., Qiu L., Shao K., Song T., Li Y. (2025). Analysis of setup error and setup efficiency in SBRT for non-small cell lung cancer via a surface-guided radiation therapy system. BMC Cancer.

